# Role of Chest CT Radiomics in Differentiating Tumorlets and Granulomas: A Preliminary Study

**DOI:** 10.3390/jcm15010210

**Published:** 2025-12-27

**Authors:** Alessandra Siciliani, Gisella Guido, Domenico De Santis, Benedetta Bracci, Benedetta Masci, Antongiulio Faggiano, Nevena Mikovic, Piero Paravani, Maurizio Martiradonna, Federica Palmeri, Chiara De Dominicis, Massimiliano Mancini, Marta Zerunian, Beatrice Trabalza Marinucci, Giulio Maurizi, Erino Angelo Rendina, Marco Francone, Andrea Laghi, Mohsen Ibrahim, Damiano Caruso

**Affiliations:** 1 Thoracic Surgery Unit, Department of Surgical Medical Sciences and Translational Medicine, Sapienza University of Rome, Sant’Andrea University Hospital, Via di Grottarossa, 1035/1039, 00189 Rome, Italy; alessandrasiciliani@gmail.com (A.S.); beatrice.trabalzamarinucci@uniroma1.it (B.T.M.); giulio.maurizi@uniroma1.it (G.M.); erinoangelo.rendina@uniroma1.it (E.A.R.); mohsen.ibrahim@uniroma1.it (M.I.); 2 Radiology Unit, Department of Medical-Surgical Sciences and Translational Medicine, School of Medicine and Psychology, Sapienza University of Rome, Sant’Andrea University Hospital, 00189 Rome, Italy; gisellaguido29@gmail.com (G.G.); domenico.desantis@hotmail.it (D.D.S.); benedetta.bracci@gmail.com (B.B.); benedetta.masci@uniroma1.it (B.M.); federica.palmeri@uniroma1.it (F.P.); dedominicischiara.1@gmail.com (C.D.D.); marta.zerunian@uniroma1.it (M.Z.); marco.francone@uniroma1.it (M.F.); 3 Endocrinology Unit, Department of Clinical and Molecular Medicine, Sant’Andrea Hospital, ENETS Center of Excellence, Sapienza University of Rome, 00189 Rome, Italy; antongiulio.faggiano@uniroma1.it (A.F.); nevena.mikovic@uniroma1.it (N.M.); piero.paravani@uniroma1.it (P.P.); maurizio.martiradonna@uniroma1.it (M.M.); 4 Pathology Unit, Sant’Andrea Hospital, University of Rome La Sapienza, 00189 Rome, Italy; 5 Department of Biomedical Sciences, Humanitas University, Via Rita Levi Montalcini 4, 20072 Pieve Emanuele, Italy; andrea.laghi@hunimed.eu; 6 Department of Diagnostic Imaging, IRCCS Humanitas Research Hospital, Via Manzoni 56, 20089 Rozzano, Italy

**Keywords:** tumorlets, granulomas, chest CT, radiomics, segmentation analysis

## Abstract

**Background:** To identify the radiomics features of both granulomas and tumorlets (TL) and to assess the potential role of radiomics in differentiating these two diseases. **Methods:** From 2013 to 2021, ninety patients who had undergone lung surgery and pre-operative chest CT evaluation, with pathologically proven granulomas or TL, were retrospectively enrolled. Two radiologists, in consensus, manually segmented the lesions on CT images. Radiomic features were then automatically extracted from these segmentations using dedicated software. The performance of CT radiomics features in differentiating TL from granulomas was tested by receiver operating characteristic curves and the areas under the curve (AUCs), calculating sensitivity and specificity. **Results:** The final population consisted of 55 patients (38 female; mean age 64 ± 14 years), 32 with TL and 23 with granulomas. Significant differences were found in 16/107 radiomic features: 3 Shape, 1 First Order, 2 Grey Level Co-occurrence Matrix (GLCM), 2 Gray Level Dependence Matrix (GLDM), 4 Grey Level Run Length Matrix (GLRLM), and 4 Gray Level Size Zone Matrix (GLSZM). Flatness and Long Run High Gray Level Emphasis showed the best performances in discriminating TL from granulomas (AUC: 0.903; sensitivity: 100%; specificity: 80%; and AUC: 0.896; sensitivity: 92.3%; specificity: 76.5%; respectively; both *p* < 0.001). **Conclusions:** Radiomics may be a non-invasive imaging tool for characterization of small lung nodules, differentiating granulomas from TL, and may play a role in preventing TL growth and its possible malignant evolution, avoiding delayed diagnosis.

## 1. Introduction

**Figure 1:**
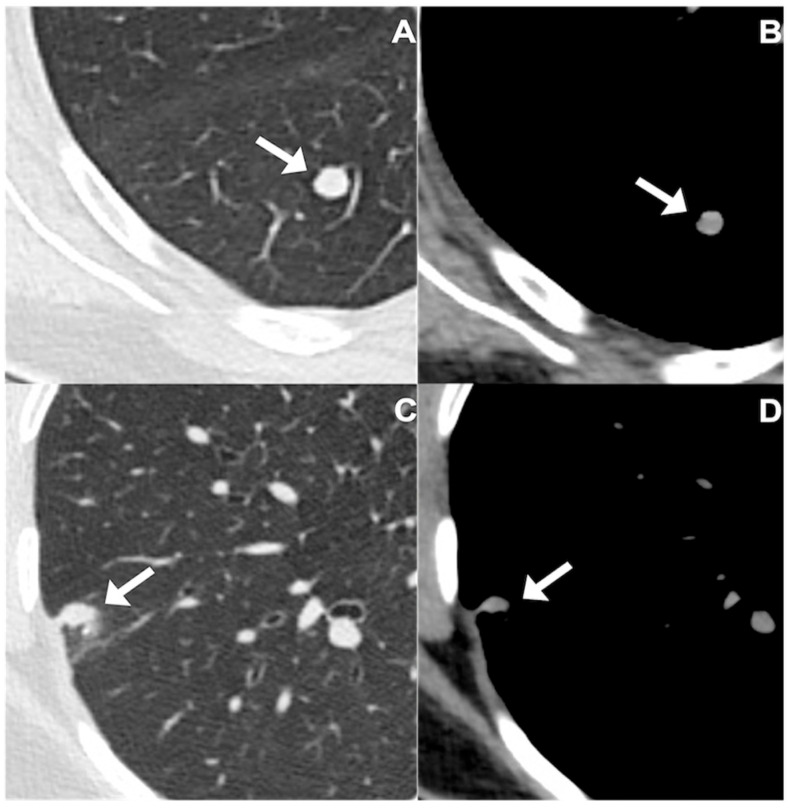
(**A**) Lung and (**B**) mediastinal window settings on a non-contrast chest CT in a 54-year-old male. The nodule (arrow) shows round shape, regular margins, no spiculation. After surgery, a pathological diagnosis of tumorlet was given. (**C**) Lung and (**D**) mediastinal window of unenhanced chest CT in 67-years-old female patient. The nodule (arrow) shows ovalar shape, feather edges, regular margins, and no spiculation. After surgery, a pathological diagnosis of tumorlet was given.

Solid pulmonary nodules are a relatively frequent finding on chest CT scans. Although the majority ultimately prove to be benign, their detection always raises concern because some of these lesions may correspond to an early and potentially curable lung malignancy. Over the past few years, significant technological innovations in multidetector CT systems, together with the increasing adoption of structured lung cancer screening programs, have resulted in a substantial rise in the identification of incidental solitary nodules. This frequent occurrence represents a recurrent diagnostic challenge for clinicians and radiologists. A comprehensive evaluation that takes into account both the patient’s clinical risk profile and the detailed CT features of each nodule—such as dimensional measurements, temporal evolution, contour characteristics, presence of calcific foci, attenuation pattern, and anatomical distribution—generally supports the estimation of the probability of malignancy and assists in selecting the most appropriate management strategy, in line with the recommendations of the Fleischner Society [1]. It is important to highlight that the 2015 edition of the World Health Organization classification of tumors involving the lung, pleura, and thymus [2,3,4] introduced for the first time preinvasive cancerous lung lesions, namely tumorlets and DIPNECH, in the section of neuroendocrine tumors of the lung [5]. Tumorlets are defined as proliferation of nodular neuroendocrine cells with a diameter < 5 mm, while DIPNECH represents an idiopathic diffuse hyperplasia of neuroendocrine cells and/or multiple associated tumorlets; to date, the preinvasive behavior of these lesions is still not fully clarified [6,7]. Furthermore, the 2021 revision of the WHO classification [8] clarified the criteria for neuroendocrine cell proliferations, specifying that lesions measuring up to 5 mm should be designated as *tumorlets*, whereas larger lesions fall into the category of *typical carcinoid*. An illustrative example of a tumorlet is presented in Figure 1. In light of these definitions, radiologists are encouraged to pay particular attention to any solid pulmonary nodule measuring 5 mm or more, carefully assessing their morphological characteristics and growth pattern. When such nodules meet the size threshold or show suspicious imaging features, referral to thoracic surgery for resection and subsequent pathological confirmation is generally recommended in order to establish the presence of true malignancy and guide appropriate clinical management.

Because surgical removal of a pulmonary nodule measuring less than 5 mm is generally not justified in the absence of a prior histopathological diagnosis, there is a clear clinical need for reliable, non-invasive diagnostic approaches that could help characterize these very small lesions. Developing imaging-based methods capable of distinguishing tumorlets from other benign conditions, such as post-inflammatory granulomas, would significantly improve decision-making, limit unnecessary surgical procedures, and ultimately contribute to a more tailored management of patients with incidentally detected small lung nodules. Radiomics enables an advanced quantitative analysis of medical images [9] providing imaging biomarkers that can indirectly reflect underlying biological characteristics such as cellular heterogeneity, molecular pathways, and even gene-expression patterns. By extracting a large number of reproducible imaging features from standard CT acquisitions, radiomic techniques may substantially support the evaluation of indeterminate pulmonary nodules. In this context, their integration into clinical practice has the potential to reduce the need for invasive diagnostic procedures and to minimize unwarranted surgical interventions, thereby optimizing patient management and risk stratification [10,11].

In the currently available literature, only a limited number of investigations have specifically addressed the potential contribution of CT-based radiomic analysis in distinguishing pulmonary neuroendocrine neoplasms from other benign pulmonary conditions [12,13]. Therefore, the primary objective of the present investigation is to characterize the chest CT radiomic features associated with both granulomatous lesions and tumorlets, and to explore whether these quantitative parameters can assist in distinguishing the two entities. By identifying radiomic patterns that may be indicative of early neuroendocrine proliferation, this approach could potentially contribute to the earlier detection of pre-cancerous alterations and facilitate more appropriate clinical decision-making.

## 2. Materials and Methods

### 2.1. Patient Population and Study Design

This retrospective observational study was in accordance with the Declaration of Helsinki. Approval was granted by the local ethics boards (CE 7031/2022) and all participants provided informed consent.

Between 2013 and 2021, ninety consecutive individuals who underwent lung surgery at Sant’Andrea University Hospital were retrospectively identified and considered for inclusion. Eligible patients were required to meet the following criteria: (a) a definitive histopathological diagnosis of either pulmonary tumorlets or granulomatous lesions, and (b) the availability of a preoperative, non-contrast chest CT examination suitable for radiomic analysis. Patients were excluded in the presence of one or more of the following conditions: (a) substantial respiratory motion artifacts affecting image quality, (b) lack of an unenhanced CT acquisition, or (c) lesions with dimensions insufficient for reliable segmentation or quantitative feature extraction.

Demographic and clinical information, such as age, sex, comorbidities, prior oncologic history, smoking habits, and final pathological report, was retrieved from institutional medical records and subsequently analyzed for the purposes of the study.

### 2.2. CT Scan Protocol and Image Reconstruction

Chest CT scans were performed in the cranio-caudal direction with patients positioned supine and instructed to maintain a deep inspiration throughout image acquisition. No intravenous contrast medium was administered for any of the examinations. All imaging was conducted on a 128-slice CT scanner (Revolution EVO, GE Medical Systems, Chicago, IL, USA). The acquisition protocol included a tube voltage of 120 kV, automated tube current modulation with a range of 100–250 mAs, a spiral pitch of 0.98, and a collimation width of 0.625 mm. Raw data were reconstructed using iterative reconstruction software (ASiR-V, 50%, GE Medical Systems) at a slice thickness of 0.5 mm, and multiplanar reconstructions were subsequently generated to allow comprehensive evaluation of pulmonary structures and nodules.

### 2.3. Evaluation and Segmentation of Chest CT Images

**Figure 2:**
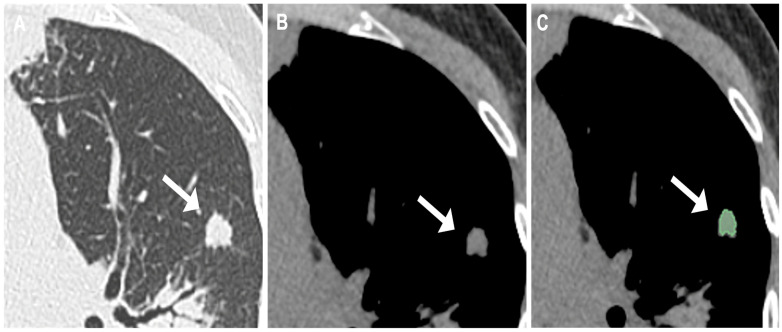
(**A**) Lung window and (**B**) mediastinal window images from a non-contrast chest CT of a 63-year-old female patient. Images show small solid nodule in left superior lobe (arrows) with pathologically proven diagnosis of granuloma. (**C**) Radiomics volumetric segmentation (highlighted in green).

DICOM datasets were exported and transferred to a multimodality workstation (Centricity Universal Viewer, version 6.0; GE Medical Systems) for further evaluation. Two thoracic radiologists, with 15 and 5 years of experience in chest imaging respectively, independently reviewed all CT examinations and performed manual segmentation of the pulmonary lesions. Each radiologist delineated the regions of interest (ROIs) separately, following a standardized segmentation protocol. In cases of disagreement between the two independent segmentations, a dedicated consensus session was held, during which discrepancies were carefully reviewed and resolved. The resulting final volumetric contour for each lesion was then used for subsequent radiomic feature extraction. Volumetric segmentation of the lesions was performed using the open-source software 3D Slicer (version 4.11.20210226, http://www.slicer.org, accessed 28 February 2021). For each lesion, a volumetric region of interest (VOI) was manually drawn on a slice-by-slice basis using the mediastinal window. Care was taken to strictly exclude adjacent pulmonary vessels, bronchi, and mediastinal structures, thereby ensuring that only the lesion itself was encompassed. This approach allowed complete coverage of the lesion volume, providing an accurate three-dimensional representation for quantitative radiomic analysis, as illustrated in Figure 2.

### 2.4. Extraction of Radiomic Features

3D Slicer Radiomics extension (PyRadiomics library, version 3.0.1 [14]) was used to extract 107 radiomic features from the consensus segmentation of the lesions. Prior to feature extraction, image preprocessing included voxel resampling to isotropic 1 × 1 × 1 mm^3^ spacing using B-spline interpolation, and intensity discretization with a fixed bin width of 25 Hounsfield units, in accordance with IBSI recommendations. Radiomic features were extracted from the mediastinal window, which was selected because it provides more stable attenuation values for small solid nodules and reduces variability related to surrounding lung parenchyma. The extracted features included first- and second-order metrics: 19 first-order statistics, 13 2D/3D shape features, 16 Gray Level Size Zone Matrix (GLSZM), 5 Neighbouring Gray Tone Difference Matrix (NGTDM), 14 Gray Level Dependence Matrix (GLDM), 24 Gray Level Co-occurrence Matrix (GLCM), and 16 Gray Level Run Length Matrix (GLRLM).

### 2.5. Statistical Analysis

Statistical analyses were conducted using MedCalc Statistical Software, version 20.013 (MedCalc Software bvba, Ostend, Belgium). Continuous variables are presented as mean ± standard deviation (SD), whereas categorical variables are expressed as absolute counts and corresponding percentages. The normality of continuous data was assessed using the Shapiro–Wilk test. Parametric variables were compared using Student’s *t*-test, while non-parametric variables were analyzed with the Mann–Whitney U test. To account for the potential inflation of type I error due to multiple comparisons, the Holm–Bonferroni method was applied as a correction strategy [15]. Receiver operating characteristic (ROC) curve analysis was performed for each significant variable in order to determine the area under the curve (AUC), along with sensitivity and specificity values. Throughout the analyses, a two-sided *p* value < 0.05 was considered indicative of statistical significance.

## 3. Results

### 3.1. Study Cohort, Patient Characteristics, and CT Findings

**Figure 3:**
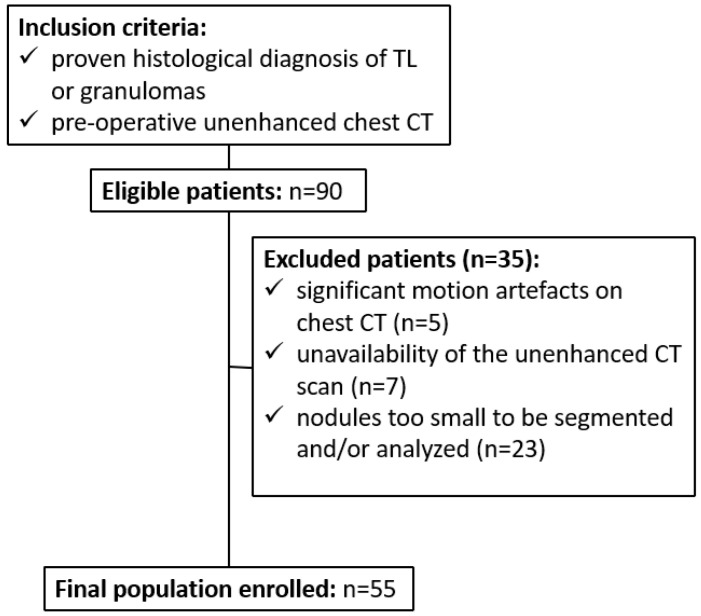
Flowchart of Patient Enrollment.

From an initial cohort of 90 patients, including 55 diagnosed with tumorlets (TL) and 35 with granulomas, a total of 35 individuals (39%) were excluded from the analysis. Specifically, 5 patients (5%) were excluded due to significant motion artifacts on CT images, 7 patients (8%) were excluded because unenhanced chest CT scans were not available, and 23 patients (25%) had pulmonary nodules that were too small to allow reliable segmentation or quantitative analysis. Consequently, the final study population comprised 55 patients, of whom 38 (69%) were female and 17 (31%) were male, with a mean age of 64 ± 14.6 years (range: 24–84 years). The enrollment process and patient selection workflow are summarized in the flowchart presented in Figure 3.

Among the final cohort, 32 patients (58%) were diagnosed with tumorlets (TL), while the remaining 23 patients (42%) had granulomas. In terms of nodule location, 13 lesions (24%) were situated in the left lower lobe, comprising 11 TL and 2 granulomas. Regarding nodule morphology, the majority—35 nodules (64%)—exhibited regular margins (23 TL and 12 granulomas), whereas 17 nodules (31%) were characterized by spiculated contours (9 TL and 8 granulomas). Comorbidities were present in 38 patients (69%), with hypertension and diabetes mellitus being the most frequently reported conditions. A total of 17 participants (31%) had a prior history of at least one neoplasm, with breast cancer representing the most common malignancy. Concerning smoking status, 24 patients (44%) were current or former smokers, while 31 patients (56%) reported no history of tobacco use. Detailed demographic, clinical, and CT characteristics of the study population are summarized in Table 1 and Table 2.

### 3.2. Radiomics Features and ROC Curves

A total of 107 radiomic features were extracted from the volumetric segmentation (VOI) of each lesion on chest CT scans. Following adjustment for multiple comparisons, 16 features were found to differ significantly between the groups, as summarized in Table 3.

**Figure 4:**
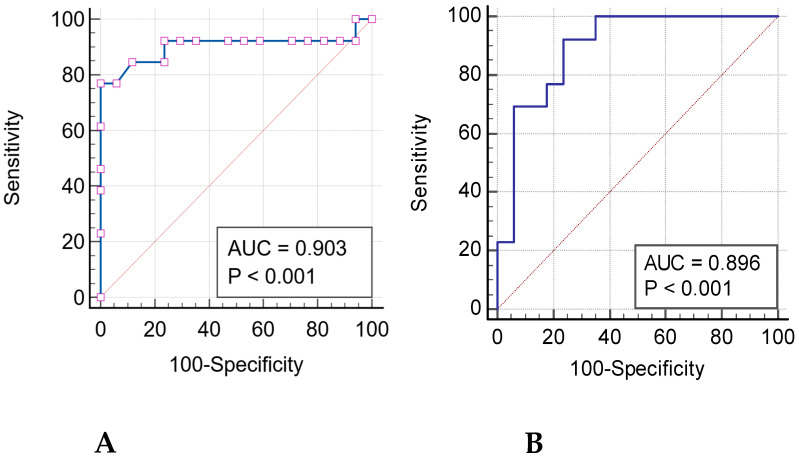
Receiver operating characteristic (ROC) curves, used to test the performance of radiomics features in discriminating tumorlets from granulomas, show the best two area under the curves (AUCs): (**A**) Flatness showed an AUC of 0.903, sensibility of 76.9%, and specificity of 100%, (*p* < 0.001); (**B**); Long Run High Gray Level Emphasis demonstrated an AUC of 0.896, sensibility of 92.3%, and specificity of 76.5% (*p* < 0.001).

In particular, among Shape features, three (Flatness, Least Axis Length, and Surface Volume Ratio) were able to significantly differentiate between two patient groups (*p* < 0.001) with the respectively performances of Flatness (AUC: 0.903, sensitivity: 76.9%, specificity: 100%, *p* < 0.001), Surface Volume Ratio (AUC: 0.835, sensibility: 100%, specificity: 58.8%, *p* < 0.001) and Least Axis Length (AUC: 0.889, sensitivity: 64.7%, specificity: 84.6%, *p* < 0.001) in discriminating TL from granulomas (Figure 4).

Among First Order features, only Range significantly differentiate TL from granulomas (*p* < 0.001) with a good performance (AUC: 0.864, sensitivity: 84.6%, specificity: 82.3%, *p* < 0.001) for TLs individuation. Significant differences were found in two GLCM features (Joint Average and Sum Average) that demonstrated significant differences between two groups (*p* < 0.001); in particular, performances in discriminating TL were shown by Joint Average (AUC: 0.828, sensitivity: 92.3%, specificity: 70.5%, *p* < 0.001) and Sum Average (AUC: 0.846, sensitivity: 84.6%, specificity: 76.5%, *p* < 0.001).

Among GLDM features, Dependence Non Uniformity and High Gray Level Emphasis were able to identify TL in significant way (*p* < 0.001), with the performances of Dependence Non Uniformity (AUC: 0.894, sensibility: 100%, specificity: 64.8%, *p* < 0.001) and High Gray Level Emphasis (AUC: 0.869, sensibility: 93.3%, specificity: 76.5%, *p* < 0.001).

Four GLRLM features (High Gray Level Run Emphasis, Long Run High Gray Level Emphasis, Run Entropy and Short Run High Gray Level Emphasis) showed significant differences between TL and granulomas (*p* < 0.001). Among them, the best performances in differentiating between TL and granulomas were reached by Long Run High Gray Level Emphasis and Short Run High Gray Level Emphasis (AUC: 0.896, sensibility: 92.3%, specificity: 76.5%, *p* < 0.001 e AUC: 0.869, sensibility: 92.3%, specificity: 76.5%, *p* < 0.001, respectively) (Figure 4).

Among GLSZM features, four (High Gray Level Zone Emphasis, Low Gray Level Zone Emphasis, Small Area High Gray Level Emphasis, and Small Area Low Gray Level Emphasis) showed significant differences between TL and granulomas (*p* < 0.001). In particular, Small Area Low Gray Level Emphasis and Low Gray Level Zone Emphasis showed the best performances at ROC curves (AUC: 0.898, sensibility: 92.3%, specificity: 70.6%, *p* < 0.001; and AUC: 0.894, sensibility: 76.9%, specificity: 88.2%, *p* < 0.001, respectively).

None of the NGTDM features showed significant differences in discriminating TL and granulomas.

## 4. Discussion

This retrospective study investigated the main CT radiomics features of TL and granulomas in 55 patients who underwent surgical resection, in order to establish the potential role of radiomics in differentiating these two entities. Statistically significant differences were found in 16 out of 107 total radiomics features extracted: 3 Shape, 1 First Order, 2 GLCM, 2 GLDM, 4 GLRLM, and 4 GLSZM. In particular, Flatness and Long Run High Gray Level Emphasis demonstrated the highest discriminative performance in univariate analysis (respectively, AUC: 0.96; sensitivity: 100%; specificity: 80%; and AUC: 0.896; sensitivity: 92.3%; specificity: 76.5%; both *p* < 0.001), reflecting differences in nodule shape and intralesional texture heterogeneity.

Few studies have investigated radiomics in differentiating pulmonary neuroendocrine tumors from other lung nodules [12,13,16]. In particular, Cozzi et al. [16] retrospectively evaluated 27 patients affected by different pulmonary neuroendocrine tumor subtypes (typical and atypical carcinoids, large and small cell neuroendocrine carcinoma) reporting a significant difference in enhanced CT in multiple first-order and higher-order radiomics features in the correlation with Ki-67 index values, while for the presence of metastases, only Skewness and Cluster Shade were significant on enhanced CT.

A study conducted by Cangir and colleagues [12] successfully implemented a radiomics model in differentiating pulmonary carcinoid tumors from pulmonary hamartomas (AUCs ≥ 0.836). While tumorlets are smaller and less aggressive than the neuroendocrine tumors studied, these works are cited to illustrate the potential of radiomics in distinguishing neuroendocrine-related lesions, providing context for our exploratory analysis.

Thuillier et al. [13] evaluated the potential role of radiomics features extracted by 18F-FDG-PET/CT in discriminating between neuroendocrine tumors and carcinomas; demonstrating that conventional PET parameters were able to distinguish between these two diseases, while radiomics did not provide additional information.

On the other side, scientific literature has previously investigated the role of radiomics features to discriminate between benign and malignant lung lesions, with good accuracy [17,18]; in particular, several studies analyzed the role of CT Radiomics in the differentiation between pulmonary adenocarcinomas and granulomas. Beig et al. [19] extracted two-dimensional texture features from both intra-nodular and peri-nodular regions to distinguish malignant from benign nodules, demonstrating that the combination of texture features extracted by both regions improved the predictive ability in distinguishing adenocarcinomas from granulomas. On the same topic, Khorrami et al. [20] analyzed radiomics features extracted within the nodules and in the adjacent area, finding a valid CT radiomic classifier for lung cancer diagnosis, able to identify suspicious or intermediate risk lung nodules.

A study conducted by Liu and colleagues [21] on 875 patients with benign or malignant pulmonary nodules analyzed radiomic features extracted from chest CT scans. Radiomics nomogram showed good performances in discrimination between benign and malignant pulmonary nodules from training (AUC: 0.836) and validation cohort (AUC: 0.809), demonstrating a potential non-invasive preoperative prediction tool for pulmonary nodule diagnosis.

Prompt differential diagnosis of pulmonary nodules is crucial to achieve early identification and consequent appropriate patient management and treatment [22]; in such scenarios, radiomics has emerged as a potential tool for assisting clinicians in routine workup [18]. In this study, sixteen radiomics features of different classes were found to be significant in differentiating between TL and granulomas.

This study presents several limitations that should be acknowledged. First, the relatively small sample size and retrospective nature of the investigation inherently restrict the statistical power and limit the generalizability of the findings. Second, although a correction for multiple comparisons was applied, the analysis was based solely on univariate statistics, without the construction of a multivariate predictive model. This approach was necessitated by the limited number of cases, which would have increased the risk of overfitting in high-dimensional modeling. Consequently, the reported AUC values may be somewhat optimistic, and no internal validation procedures, such as cross-validation or bootstrapping, were performed. Third, inherent limitations of radiomic analysis remain, including the potential subjectivity associated with manual lesion segmentation, variability in CT acquisition parameters, and the absence of an independent validation cohort to confirm the robustness of the results.

It is well recognized that the reliability and predictive accuracy of radiomic analyses are strongly dependent on the size and quality of the available dataset. Consequently, further prospective studies involving larger patient cohorts are warranted to validate these preliminary findings. The ultimate goal is to develop a robust radiomics model that can be reliably tested through both internal and external validation [23], ensuring its reproducibility and practical applicability in routine clinical practice.

## 5. Conclusions

Radiomics may serve as a non-invasive tool to support the characterization of small lung nodules, helping to differentiate between TL and granulomas, and potentially aiding clinical decision-making by identifying nodules that may require closer monitoring or further investigation.

## Figures and Tables

**Table 1:** Patient data.

Patient Data	Total Patients (n = 55)	Tumorlets Group (n = 32)	Granulomas Group (n = 23)
Mean age	64 ± 14.6	68 ± 10.2	58 ± 17.7
Years (range)	24–84	43–80	24–84
Male-to-female ratio	17–38 (31%)	6–26 (19%)	11–12 (48%)
Smokers	24 (44%)	12 (38%)	12 (52%)
Comorbidities	38 (69%)	26 (81%)	12 (52%)
Previous neoplasm	17 (31%)	7 (22%)	10 (43%)

**Table 2:** CT findings.

Nodules CT Findings	Total Patients (n = 55)	Tumorlets Group (n = 32)	Granulomas Group (n = 23)
Regular margins	35 (64%)	25 (78%)	10 (43%)
Irregular margins	20 (36%)	7 (22%)	13 (57%)
Spiculations	17 (31%)	2 (6%)	15 (65%)
**Location**			
LLL	13 (24%)	11 (34%)	2 (9%)
LUL	11 (20%)	6 (19%)	5 (22%)
RUL	11 (20%)	7 (22%)	4 (17%)
RLL	11 (20%)	5 (16%)	6 (26%)

**Table 3:** Radiomic features comparison and ROC curve analysis.

Radiomics Features	TL vs. Granulomas	ROC CURVE ANALYSIS			
**SHAPE**	***p*** **Value**	**Sensitivity**	**Specificity**	**AUC**	***p*** **Value**
Flatness	<0.001	76.92	100	0.903	<0.001
Least Axis Length	<0.001	64.71	84.62	0.721	0.003
Surface Volume Ratio	<0.001	76.92	88.24	0.894	<0.001
**FIRST ORDER**					
Range	<0.001	84.62	82.35	0.864	<0.001
**GLCM**					
Joint Average	<0.001	92.31	70.59	0.828	<0.001
Sum Average	<0.001	84.62	76.47	0.846	<0.001
**GLDM**					
Dependence Non Uniformity	<0.001	84.62	82.35	0.894	<0.001
High Gray Level Emphasis	<0.001	92.31	76.47	0.869	<0.001
**GRLRM**					
High Gray Level Run Emphasis	<0.001	92.31	76.47	0.869	<0.001
Long Run High Gray Level Emphasis	<0.001	92.31	76.47	0.896	<0.001
Run Entropy	<0.001	100	64.71	0.885	<0.001
Short Run High Gray Level Emphasis	<0.001	92.31	76.47	0.869	<0.001
**GLSZM**					
High Gray Level Zone Emphasis	<0.001	92.31	82.35	0.878	<0.001
Low Gray Level Zone Emphasis	<0.001	76.92	88.24	0.894	<0.001
Small Area High Gray Level Emphasis	<0.001	92.31	82.35	0.878	<0.001
Small Area Low Gray Level Emphasis	<0.001	92.31	70.59	0.898	<0.001

## Data Availability

Data are contained within the article.

## Abbreviations

The following abbreviations are used in this manuscript:

TL	Tumorlet
AUC	Area Under the Curve
GLCM	GreyLevelCo-occurrenceMatrix
GLDM	GrayLevelDependenceMatrix
GLRLM	GreyLevelRunLengthMatrix
GLSZM	GrayLevelSizeZoneMatrix
